# Dataset on reproductive status of ovary mud crab at different salinity levels

**DOI:** 10.1016/j.dib.2019.104426

**Published:** 2019-08-28

**Authors:** Adnan Amin-Safwan, Mohd Pauzi Mardhiyyah, Mohd Affendi Izzah-Syafiah, Harman Muhd-Farouk, Hidayah Manan, Hairul Hafiz Mahsol, Musa Nadirah, Mhd Ikhwanuddin

**Affiliations:** a Institute of Tropical Aquaculture and Fisheries Research, Universiti Malaysia Terengganu, 21030 Kuala Nerus, Terengganu, Malaysia; b Impact Assessment Research Division, Fisheries Research Institute Batu Maung, 11960 Batu Maung, Pulau Pinang, Malaysia; c Institute for Tropical Biology and Conservation, Universiti Malaysia Sabah, Jalan UMS, 88400 Kota Kinabalu, Sabah, Malaysia; d Faculty of Fisheries and Food Sciences, Universiti Malaysia Terengganu, 21030 Kuala Nerus, Terengganu, Malaysia; e STU-UMT Joint Shellfish Research Laboratory, Shantou University, Guangdong 515063, China

**Keywords:** Aquaculture, Histology, Ovarian maturation stages, Salinity, *Scylla*

## Abstract

This article investigated how crabs responded to different culture salinities through ovarian maturation stages using combination of external morphology (ovarian coloration and gonadosomatic index), and histological assessment (oocyte structures and diameter sizes). A total of sixty immature crabs were sampled from coastal water of Setiu Wetlands, Kuala Nerus, Terengganu, Peninsular Malaysia, and were introduced to limb autotomy technique in order to induce molt. Crabs were reared until successfully molted, and leaves prior to hardened shell, before proceed with salinities acclimatization prior to salinity treatments (10, 20 and 30 ppt). Five crabs were randomly selected every 15 days throughout 60-day of culture (Day 15, 30, 45 and 60) for the assessment. The different between each ovarian maturation stages was recorded based on the color appearances, and Kruskal-Wallis analysis were done between gonadosomatic index and oocyte diameter sizes with different salinity treatments. Part of the data is associated with the recent articles [1], [2] and provided here as raw data of Supplementary materials.

**Table:** Specifications Table

Subject area	Agriculture and Biological Sciences; Aquaculture
More specific subject area	Fundamental biology; Reproductive Biology
Type of data	Tables and figures
How data was acquired	Samples dissection and histological procedures
Data format	Raw and analyzed
Experimental factors	Immature crabs from wild were sampled, proceed with limb autotomy procedure, and cultured until complete molted. After molted, crabs were acclimatized, and cultured at different salinity treatments (10, 20 and 30 ppt) for 60-day study period
Experimental features	Determination of ovarian maturation stages through external morphology (ovarian coloration and gonadosomatic index, GSI) and histological assessment (oocyte structures and sizes)
Data source location	Setiu Wetlands, Terengganu, Peninsular Malaysia (5°31′23.1″N 102°55′56.1″E) and Institute of Tropical Aquaculture and Fisheries Research (AKUATROP), Universiti Malaysia Terengganu (UMT)
Data accessibility	Data was provided in this article
Related research article	Amin-Safwan, A., Muhd-Farouk, H., Mardhiyyah, M.P., Nadirah, M., and Ikhwanuddin, M. Does water salinity affect the level of 17β-estradiol and ovarian physiology of orange mud crab, *Scylla olivacea* (Herbst, 1796) in captivity? J. King Saud Uni. – Sci. (in press). https://doi.org/10.1016/j.jksus.2018.08.006[1] Amin-Safwan, A., Muhd-Farouk, H., Nadirah, M., and Ikhwanuddin, M. Effect of water salinity on the external morphology of ovarian maturation stages of orange mud crab, *Scylla olivacea* (Herbst, 1796) in captivity. Pakistan J. Biol. Sci. 19 (2016): 219–226. https://doi.org/10.3923/pjbs.2016.219.226[2]

**Table:** 

**Value of the data** • The high nutritional value and market demands for mature and gravid female mud crabs [1], [2], [3], [4], [5], [6], [7] has triggered the present study on ovarian maturation of mud crabs. • Ovarian maturation might be one of the potential indicators [8], [9] on how cultured animals adapt to fluctuating environments such as water salinity. • The effects of water salinity on ovarian maturation stages (through external morphology and histological assessment) could be useful for other researchers investigating on adaptive evolution in climate change scenario for future study. • Such data can be useful in developing baseline for culture purposes in captivity and hatchery practice in the future [10], [11], [12], [13].

## 1 Data

Included in this article are the raw data, descriptive data (means), and Kruskal-Wallis test on the effects of different water salinities on ovarian maturation stages of the orange mud crab, *Scylla olivacea*. The shared data are recordings from the works starting from the sampling site, which involved the mud crabs sampling activities, proceed with hatchery phases (rearing periods for limb autotomy procedure, acclimatization period, and salinity treatments), and laboratory works, where the assessment activities were performed – external morphology and histology determination; percentage of ovarian maturation based on coloration (Sheet 1), gonadosomatic index (Sheet 2), oocyte diameter sizes (Sheet 3), and characterization of external morphology and microscopic observation on each maturation stage (Sheet 4).

## 2 Experimental design, materials, and methods

### 2.1 Sampling

**Fig. 1:**
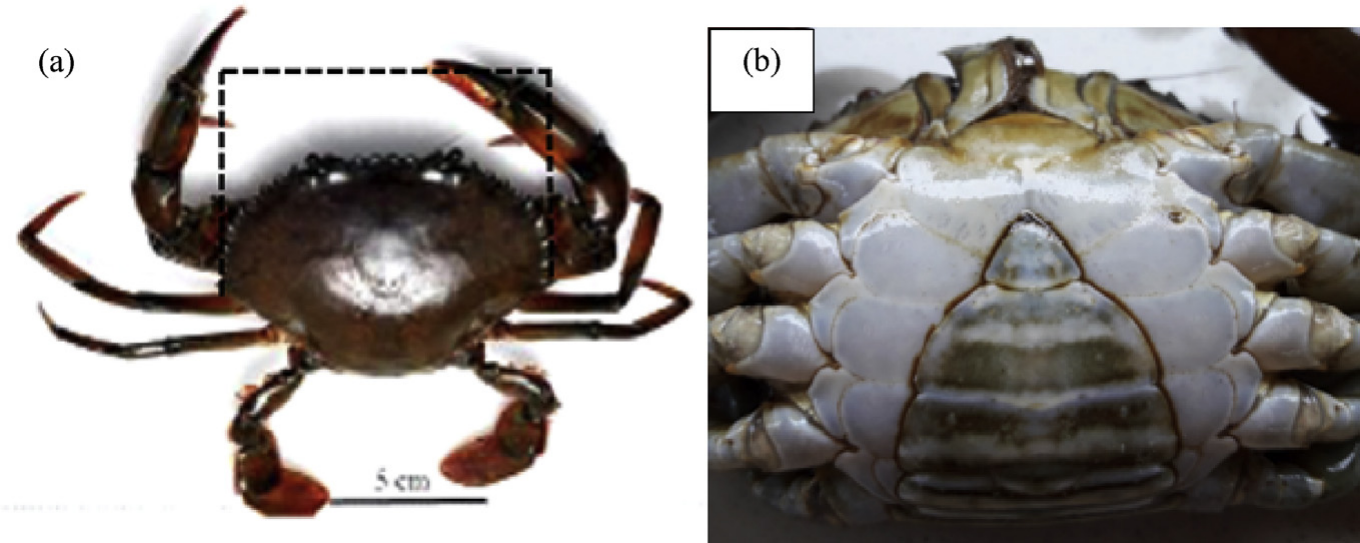
Indicator for selection of samples during present study, (a) carapace width, the distance between the tips of the 9th anterolateral spine of the crab carapace (less than 9.06 cm), and (b) small and pale abdominal flap for immature female *S. olivacea* sampled from Setiu Wetlands, Terengganu, Malaysia.

**Fig. 2:**
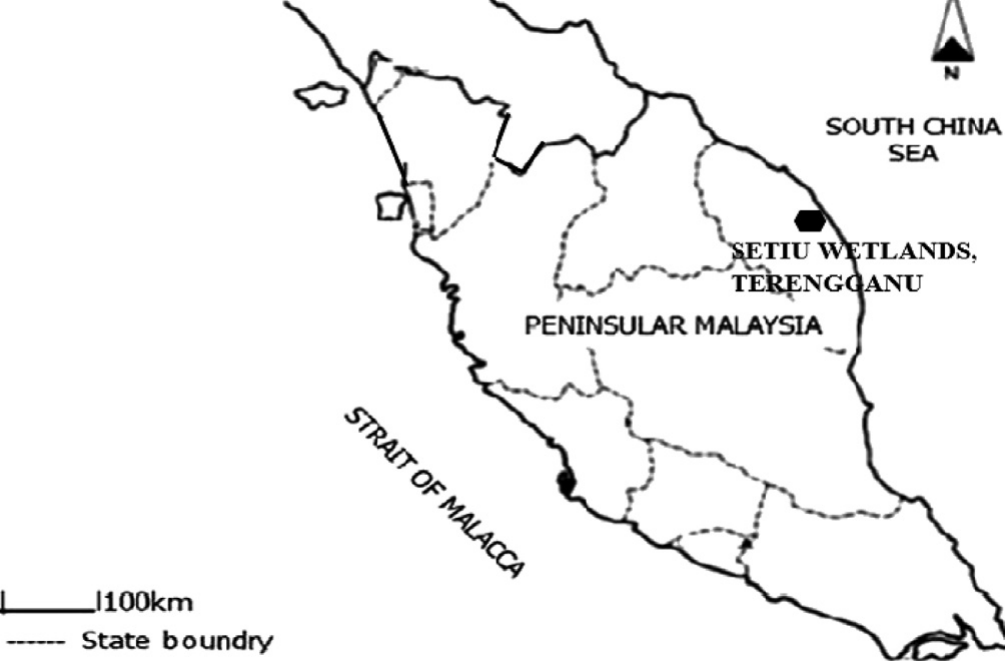
Sampling site, Setiu Wetlands, Terengganu, Malaysia. (5°31′23.1″N 102°55′56.1″E).

A total of sixty immature female *Scylla olivacea* (carapace width, CW less than 9.06 cm; small and pale abdominal flap) (Fig. 1) were sampled from Setiu Wetlands Mangrove Forest, Terengganu, East Coast of Peninsular Malaysia (5°31′23.1″N 102°55′56.1″E) (Fig. 2). Conventional rectangular collapse crab pots (dimension: length x width x height = 87 cm × 56 cm x 30 cm; mesh size = 1 cm) with openings at the middle of both end sections were used during sampling section [9]. Crab pots were deployed during the low tide in the evening (1600 h) and collected during subsequent low tide in the morning of the next day (0800 h). Only immature female crabs were chosen, and the crab samples were then transferred to the Institute of Tropical Aquaculture Marine Hatchery, Universiti Malaysia Terengganu for subsequent analysis.

The initial body weight (BW) and carapace width (CW) of each crab were measured and recorded. BW was measured using a digital balance (accuracy: 0.01 g; Shimadzu model, Japan), whereas carapace width, the distance between the tips of the 9th anterolateral spine of the crab carapace [1], [5] was determined using a six-inch liquid crystal display (LCD) digital Vernier caliper (accuracy: 0.01 cm; Kingsmart brand, Hong Kong). The crabs were then proceed with limb autotomy procedure, placed individually in a container, fed with chopped yellowstripe scad fish, *Selaroides leptolepis*, at 10% of their BW twice daily (at 0900 and 1700 h), and were maintained until completed molted.

### 2.2 Limb autotomy

The chelipeds (claws) and pereiopods (walking legs) were cast off, leaving only the pleopods (swimming legs) for the crabs’ movement [14]. The autotomized crabs were placed individually in a container, with ambient salinity (28–32 ppt), maintained temperature (27–29 °C), moderate aeration, dark light intensity (tanks were fully covered with black net, except for feeding activities), and 100% water exchange every two days. The crabs were fed with chopped scadfish, *Selaroides* spp., at 10% of their body weight twice daily (at 0900 and 1700 h) [1], [2] for observation of the molting activity.

### 2.3 Treatments

**Fig. 3:**
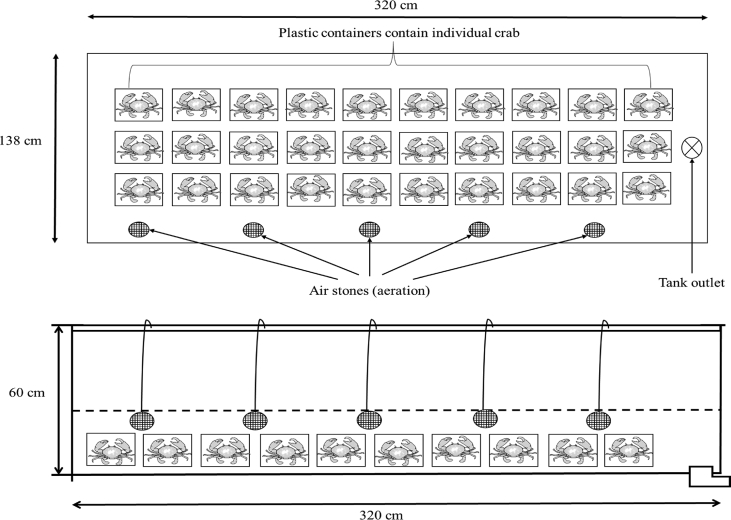
Upper view and side view of the culture tank set up for each salinity treatment regime (10, 20 and 30 ppt) of *S. olivacea* during present study.

Twenty newly molted (mature size with immature ovary – Stage 1) [3] were reared in Treatment 1, T1 (10 ppt), Treatment 2, T2 (20 ppt), and Treatment 3, T3 (30 ppt) water salinity [1] (Fig. 3). First, crab samples were acclimated until salinity treatments were achieved according to Amin-Safwan et al. [1]. Water parameters includes dissolved oxygen (DO), temperature, conductivity, pH, oxidation-reduction potential, and salinity were maintained and monitored daily using a YSI 556 MPS multi-probe meter (YSI Incorporated, Ohio) and refractometer (ATAGO, Japan). The crabs were fed with chopped *S. leptolepis*, at 10% of their body weight twice daily (at 0900 and 1700 h), and 100% water changes were performed every two days.

Every 15 day throughout the 60-day study period (Day 15, 30, 45 and 60), five crabs were randomly selected for dissection and evaluation of ovarian maturation stages through coloration, gonadosomatic index, and ovaries were fixed in Davidson's solution for further histological assessment to determine the oocyte structures and diameter sizes [1], [3], [4].

### 2.4 Data analysis

The ovarian maturation stages were distinguished by the coloration of the ovary referred to the previous studies; where translucent to creamy white (Stage 1), yellowish (Stage 2), pale to dark orange (Stage 3), and dark orange to reddish orange (Stage 4) [4], [5], [6], [7].

As for the gonadosomatic index, GSI, the calculation was as follows [2], [4]:

$$\text{Gonadosomatic}\;\text{index}=\frac{\text{Ovary}\;\text{Weight }\left(\text{g}\right)}{\text{Body}\;\text{Weight }\left(\text{g}\right)}\text{x}100$$

Meanwhile, for oocyte diameter size and structure analysis, we referred to Amin-Safwan et al. [1], where 100 oocytes for each sample (x-axis) were measured using the advanced microscope (Leica Microsystem, Wetzlar GmbH, DM LB 2, Germany). The final reading of the oocyte diameter size was averaged accordingly to respective salinity treatments.

The collected data were analyzed using Kruskal-Wallis test analysis through the SPSS software (Version 22.0 for Windows; Statistical Package for the Social Science Inc. Armonk, NY: IBM Corp.), available at https://www.ibm.com/products/spss-statistics.
